# Self-serving incentives impair collective decisions by increasing conformity

**DOI:** 10.1371/journal.pone.0224725

**Published:** 2019-11-14

**Authors:** Sepideh Bazazi, Jorina von Zimmermann, Bahador Bahrami, Daniel Richardson

**Affiliations:** 1 Independent researcher, London, United Kingdom; 2 Department of Experimental Psychology, University College London, London, United Kingdom; 3 Institute of Cognitive Neuroscience, University College London, London, United Kingdom; University of Sheffield, UNITED KINGDOM

## Abstract

The average judgment of large numbers of people has been found to be consistently better than the best individual response. But what motivates individuals when they make collective decisions? While it is a popular belief that individual incentives promote out-of-the-box thinking and diverse solutions, the exact role of motivation and reward in collective intelligence remains unclear. Here we examined collective intelligence in an interactive group estimation task where participants were rewarded for their individual or group’s performance. In addition to examining individual versus collective incentive structures, we controlled whether participants could see social information about the others’ responses. We found that knowledge about others’ responses reduced the wisdom of the crowd and, crucially, this effect depended on how people were rewarded. When rewarded for the accuracy of their individual responses, participants converged to the group mean, increasing social conformity, reducing diversity and thereby diminishing their group wisdom. When rewarded for their collective performance, diversity of opinions and the group wisdom increased. We conclude that the intuitive association between individual incentives and individualist opinion needs revising.

## Introduction

A group of individuals can have the ability to perform more effectively than any individual alone. Group members, each with independently acquired information, share and enhance their knowledge through social interactions, which allows groups to collectively solve cognitive problems in ways unavailable to isolated individuals [1]. This widespread phenomenon is known as collective intelligence or the ‘wisdom of the crowd’ effect [2], and has the potential to solve many important and complex decision-making problems in society, such as predicting economic fluctuations or political changes [3,4].

The first empirical illustration of collective intelligence was in 1907 using data from a country fair ox weight-judging contest [5]. The average of people’s estimates resulted in an accurate approximation of the ox’s weight, consistently better than the best guess of any individual. When individuals’ estimates, whilst inaccurate, are independent and symmetrically distributed around the correct answer, their individual errors cancel out each other when averaged, resulting in an accurate group answer [6]. Collective intelligence has more recently been demonstrated in various problem-solving experiments [7–9], and is increasingly being used to solve real-world problems, such as predicting stock prices [10], reducing climate change [11], or creating an online encyclopaedia [12]. However, it can be vulnerable to groupthink [13], confirmation bias [14], and emotional contagion [15], which together can instead give rise to ‘the madness of crowds’ [16]. Under certain social conditions, groups do not out-perform individuals; instead collective decisions become more extreme or less wise than choices group members would take alone.

One of the ways for a crowd to be ‘wise’ is the presence of diversity of judgements among group members. This ensures enough within-group variance and limited bias for an accurate collective response to emerge [2]. In real-world situations individuals operating in social situations frequently adjust their views to those of others [17,18], often aggregating opinions through social interactions. This reduction in group diversity through the alignment of opinions has previously been discussed in terms of social conformity [19], with potentially negative effects on the wisdom of the crowds. Consistent with this, Lorenz *et al*. (2011) showed that the wisdom of the crowd effect is undermined by social influence in a group estimation task. In their experiment, participants answered general knowledge questions. Group performance was less accurate whenever individuals could observe others’ estimates, or the group average. Ironically, although collective performance deteriorated when social information was presented, individuals exhibited more confidence in their answers’ accuracy than when social information was absent [20]. This suggests that by considering available social information, individuals adopt a suboptimal *collective* decision-making strategy. Conversely, when people are trying to maximise their own *individual* gains (such as individual accuracy), using social information can be beneficial, but would be detrimental on the collective performance [20].

The findings by Lorenz *et al*. (2011) convincingly demonstrate that social influence can erode collective intelligence, and lead us to the notion that using social information may provide individual gains, but the precise role of motivation and reward in conforming to social influence remains unclear.

The effect of incentives on the wisdom of crowd phenomena have been investigated theoretically in management science, using so-called "prediction contests" where individuals are competing with other group members for prizes, rewarded to those with the most accurate answers [21–23]. These studies show that rewards based on *relative* performance of individuals encourages individuals to discount public signals (information that everybody knows) preventing the crowd from suffering from “public information bias” and creating accurate crowd forecasts. In contrast, under individualised payoff schemes (the closer to the truth, the more money one can earn) people should incorporate the public signal because it makes their final estimate more accurate. The likely negative impact of individualised payoffs on the collective performance (by reducing diversity of opinions) however, has yet to be shown empirically.

Other theoretical studies suggest that maximizing the wisdom of the crowd is best achieved by an individualised payoff scheme that rewards agents for the accuracy of their own response and of a minority opinion, as this produces optimal group diversity [24,25]. This is not only consistent with Adam Smith’s idea of *homo economicus*, but also with the popular intuition that individual incentives promote innovative, outside-the-box thinking and solutions, which could collectively provide group wisdom. Yet, these studies ignore the non-trivial relationship between payoff structure and social information: inferring what counts as innovative requires individuals to understand which opinions are (or are not) of the minority. Furthermore, almost all collective decisions are made in the context of social interactions [26]. Individuals observe others, discuss opinions, and actively seek others’ thoughts and plans. As such, the assumption of independence of opinions in theoretical models that promote diverse ‘every man for himself’ [27] incentive structures is very likely problematic. The question of whether incentive structures (either individual, as in these studies, or collective) influence collective intelligence as these somewhat simplifying theoretical models assume [24,25] remains unanswered.

Here we experimentally examine a direct comparison between individual versus collective payoff structures and their impact on social conformity, specifically how they affect collective intelligence in a group decision-making context. We propose that incentive structure can explain how some groups exhibit diversity of opinion and wisdom, while other groups exhibit conformity and erroneous judgement. By explicitly exploring the benefits group members can gain under varying social conditions, we can begin to understand the motivational mechanisms that may promote or discourage conformity and their consequences on group decision-making.

Following past research on collective wisdom (Lorenz et al., 2011), we predict that the presence of social information will increase group error. Our key novel hypothesis, however, is that this effect will depend upon the nature of the incentive structure. We hypothesise that when group members provide estimates, knowing that they will be rewarded as individuals, they will copy others to maximise their individual gains [20–23,28]. Consequently, they will become less diverse and therefore less accurate as a group. Conversely, when rewarded for the performance of the group, copying others would reduce the diversity and independence of opinions, leading to poor group performance. Hence, under a collective payoff structure, we predict that individuals will maintain the diversity of their opinions, despite the presence of social information. Group accuracy, and therefore the likelihood of individuals receiving any reward increases, thus individuals’ responses will remain both diverse and collectively wise.

## Materials and methods

The study was approved by Birkbeck-UCL NeuroImaging (BUCNI) Ethics Committee, and UCL Psychology and Language Sciences Ethics Committee (Ethics Project name: Neurobiological basis of decision making in the human brain Ethics approval number: fMRI/2012/010). Written consent was obtained from participants.

**Fig 1:**
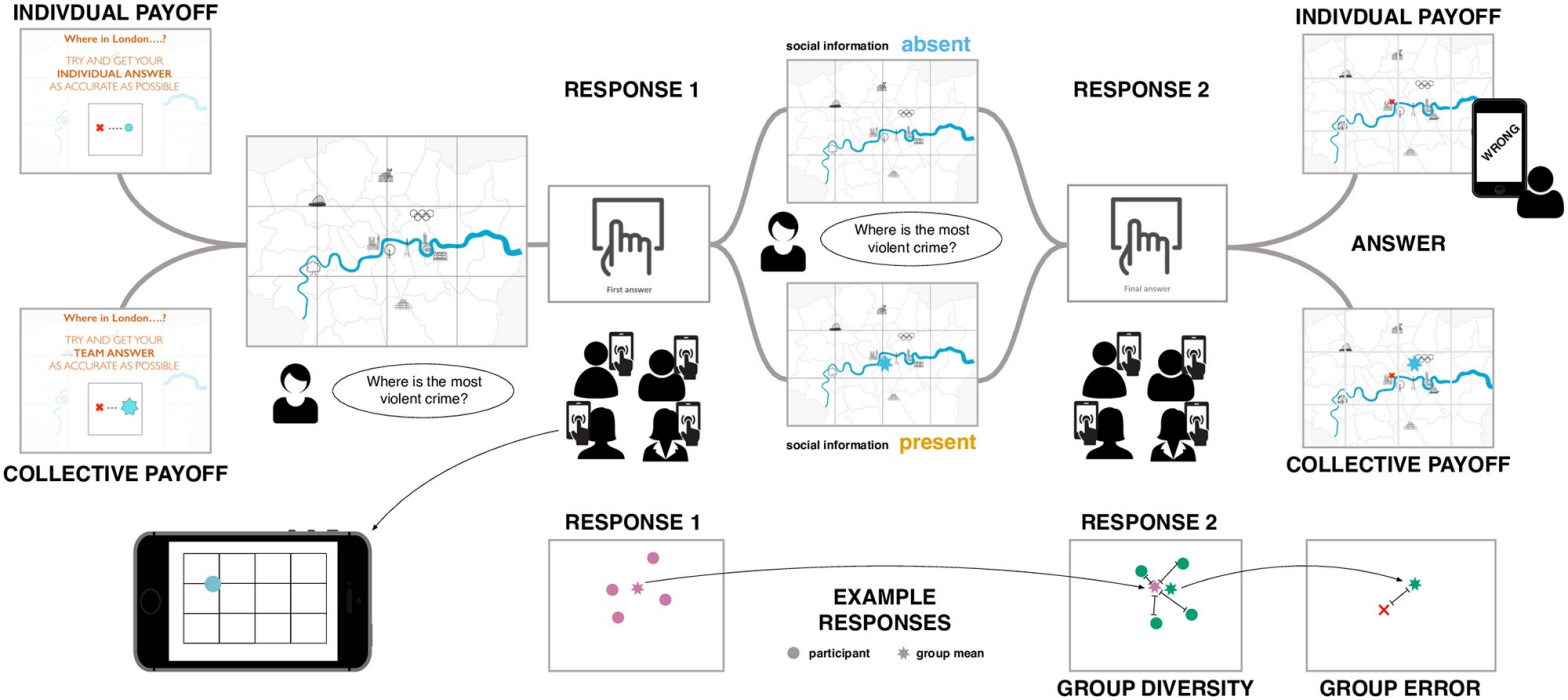
Schematic of the experiment design and operationalisations. At the start of each trial, groups were told if the trial was in the collective or individual payoff condition. Then on the central display, a map of London was presented for five seconds. A question relating to a location in London was also shown above the map and read aloud by the experimenter. The map disappeared and participants were prompted to provide their first answer, Response 1, by moving their dot on their device’s screen to give their answer. Then the map and question were displayed again for five seconds, and the question was read out loud once more. During this time participants were not permitted to move their dots. In the trials where social information was present, a star representing the position of the group’s mean Response 1 was additionally displayed on the map. Participants were then asked to provide their final answer, Response 2. Finally, the correct answer was revealed. In individual payoff condition trials, participants were informed with a feedback message on their mobile device whether their dot was in the same grid location as the correct answer. In collective payoff condition trials, participants were able to see the group’s mean Response 2 position on the shared display. A video of an example trial is available in SI (S1 Video). Example responses illustrate how group diversity and group error were operationalised. For group diversity, this was the distance between with group's mean position at Response 1, and their individual responses at Response 2. Group error was operationalised as the distance between the group mean response at Response 2 and the correct answer.

We examined individual and group behaviour in a general knowledge spatial estimation task called “Where in London?”. We used an innovative software platform, called *The Hive* [29], which allowed us to capture both individual and group behaviour, and experimentally control the information that participants received about each other. Fig 1 presents a schematic of the experiment, and a video of an example trial is available in the Supporting Information (S1 Video).

### Participants

Participants (adults, aged 18+ years old) were recruited from the University Subject Pool between June 2016 and April 2017 (by posting an advert on the University’s participant recruitment portal). We chose to use Bayesian methods to analyse our data, in which formal estimations of power and sample size do not play the same role as in frequentist statistics. Pilot work using *The Hive* software [29] suggested that good evidence for various effects of social information and decision-making could be obtained with around 120 participants. Our recruitment goal was to run between 120 and 150 people in groups of between 4 and 8 people. Following no-shows we ended up with 141 individuals in 23 different groups (70 individuals were in the individual payoff and 71 in the collective payoff treatment). Of those that declared, the mean age of our participants was 25.4 years (SD = 10.5) and 94 were female. The recruitment portal consists of participants from the general public, thus our sample can be considered representative of a larger population. All trials took place at the Department of Psychology, University College London.

All participants gave informed consent, were fully debriefed and received payment equivalent to minimum wage for their participation only. Additional payment (reward) could be received by the individual winner of the prize draw (see below), following the outcome of their performance during trials.

### Apparatus

On their laptop, the experimenter had two browser windows navigated to *The Hive* website. One was a screen that only they could see, which allowed them to control the experiment. The other was the display screen that was projected at the front of the room for everyone to see and presented experimental stimuli.

Using a browser on their smart phone or tablet provided to them, participants also visited *The Hive* website. On their device, they saw a dot that could be dragged around. On the display, visible to everyone, there was a corresponding dot for each participant. Participants’ dots were only visible on the shared display prior to experimental trials. Depending on the experimental condition, however, a star shape representing the mean of all participants’ dot locations could be shown on the display (in the social information present condition) or not (social information absent condition). Participants’ dot locations were continuously recorded by *The Hive* system throughout the experiment.

Experiments were conducted in a seminar room where participants were seated around a table all facing a projection screen (58 x 100 cm) at a distance of approx. 2 to 4.25 m.

### Procedure

Participants logged on to *The Hive* website using a code number specific to the experimental session. The display showed a welcome screen, and participants familiarized themselves with the practice of moving a dot on their device and seeing a corresponding dot move on the display. The experimenter then began a short practice session in which they gave spatial answers to questions. The experimenter explained that participants would be provided entry into a prize draw for money, and how participants would be rewarded, i.e., either according to the (*non-relative*) accuracy of their individual answer (individual payoff), or that of the group answer (collective payoff). Individuals would be entered into a prize draw for every trial where their performance was accurate in the individual payoff scheme, and for every trial where the group performance was accurate in the collective payoff scheme. The experiment session then began, lasting approximately 25 minutes. Participants were then thanked and debriefed.

### Stimuli

Groups were shown a schematic map of London projected on a large shared display at the front of the room. The map showed the river Thames, the outlines of the boroughs, and ten icons representing important landmarks. A 3x4 grid was overlaid on the display. Participants answered a series of questions about locations in London by moving the dot on the screen of their smart phone or tablet to where they thought was the correct location corresponding to the map presented on the display screen (see S1 Fig for a full list of questions asked and the answers). During the practice session, participants took approximately 5 minutes to familiarize themselves with the map.

#### Design

Participants answered 16 questions probing their knowledge of London. These trials were divided into four blocks in which we manipulated the payoff (individual/collective) and social information (present/absent) conditions in a 2 x 2 experimental design. Four different orders of the blocks and questions were created and counterbalanced across groups, so that each question appeared equally in each condition combination.

At the start of each trial, participants were reminded whether they were in the individual or collective payoff condition. Then they saw the map of London with a question above the map, which was also read aloud by the experimenter. After five seconds, the map disappeared and participants gave their first response (Response 1) by moving the dots on their devices (a corresponding 3 x 4 grid to that shown on the display was visible on participants’ devices). Participants could not see each other’s dots on the shared display. Then the map and the question were presented again for five seconds. Participants were not allowed to move their dots at this stage. In the experimental condition where social information was present, the mean location of all the group’s Response 1 was shown as a star on the display map. The mean group response was displayed to participants rather than their individual answers because displaying many dots would lessen the effect of the social context, requiring group members to accumulate the social information themselves that may be erroneous. In addition, the average position removes all the variance information from group members, and so presents the group as being more unified than they might actually be, thus provides the strongest social effect. In the social information absent condition, participants were shown only the map with no star. The map then disappeared, and all participants then answered the question again (Response 2). Finally, the map and correct answer were presented to everyone. In the individual payoff condition, the participants were told via a text message on their devices if their Response 2 answer was in the correct grid of the map. In the collective payoff condition, participants were shown the star representing their mean Response 2 on the shared display, and could see how close it was to the correct answer.

### Analysis

Group diversity was operationalised as the distance between the point representing the mean of participants’ first responses and each participant’s final response. It provided a measure of how much participants were influenced by others’ responses (as reported in the SI, we also calculated group diversity relative to the mean of the final group response, and it provided the same pattern of results and strength of evidence). Group error was operationalised as the distance between the mean location of the group’s final response, and the correct answer. It provided a measure of their group wisdom.

In this experiment, there were multiple sources of variance at the group and individual level, and so we employed a mixed-model approach. We used Bayesian hierarchical mixed models because Bayesian analyses are able to overcome some of the problems associated with null hypothesis testing [30,31].

For our measure of group error, we used fixed effects for social information and reward conditions. To account for differences due to particular participants and particular questions, we used random effects in the model each with random intercepts. In the group diversity measure, we used an additional random effect to account for the fact that each individual was nested in a particular group.

**Fig 2:**
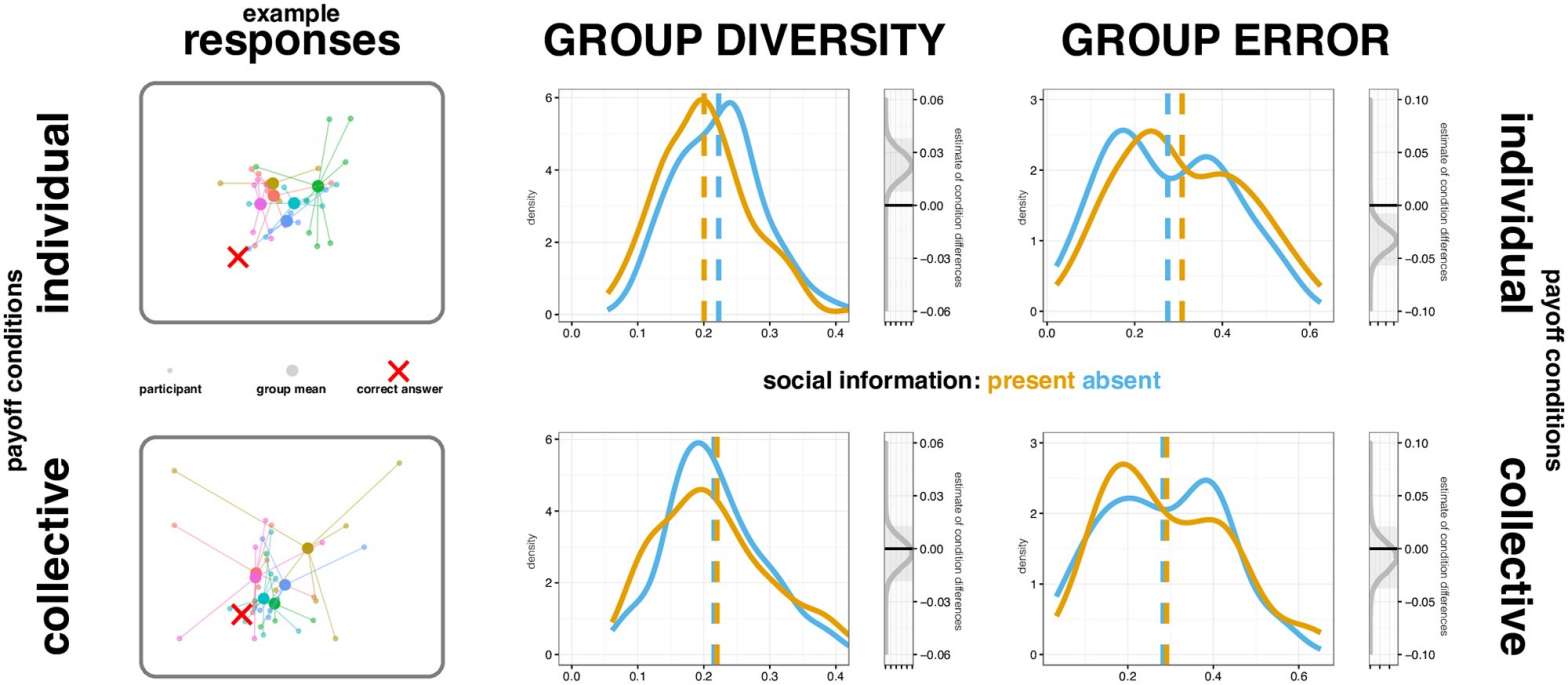
Group diversity and group error. Analysis and example response data in individual (top row) and collective (bottom row) conditions. In the left column, participants' responses to an example question: 'where do most Korean people live in London?' are shown by small dots. This trial was from the social information present condition. Dots are coloured by groups and lines join them to the group mean response, shown as a large dot. Group diversity is analysed in the centre column, and group error on the right (all questions and trials). In each density plot, solid lines show the distributions of our data when social information is present (yellow) and absent (blue), with means given by dotted lines. These distributions highlight group diversity and error for individual and collective pay-off groups; when rewarded for the accuracy of their individual responses, participants converged to the group mean, displaying reduced group diversity and increased group error. To the right of each data distribution, grey lines show the Bayesian posterior distribution for the difference between social information conditions within each payoff condition, with the 95% credibility intervals shaded in grey.

We used R (version 3.3.1; [32], the rstanarm package [33] for Bayesian analysis, and the psycho package to help interpret our models [34] and express our results in terms of the probabilities of there being main effects and interactions between conditions. From 4000 simulations, we generated estimates of the posterior distributions of the model parameter coefficients, which quantify the strength of the evidence that each experimental condition influenced behaviour. These posterior distributions are plotted in Fig 2 to the side of the observed distributions. 95% credibility intervals for these estimates are shown as grey boxes in Fig 2. If 95 credibility intervals for a parameter do not include zero, this can be interpreted as strong evidence that it had an effect. Below we report the Maximum Probability of Effect (MPE), which is the probability that the effect is positive or negative (depending on the median’s direction). In other words, the MPE directly quantifies the probability that the experimental condition had an effect on behaviour.

The Bayesian approach favours quantifying the strength of evidence in this way, rather than simply reporting whether or not an (arbitrary) threshold of significance has been passed. Having said that, researchers generally suggest that an MPE of above 90% or 95% can be thought of as ‘strong evidence’. In addition to these Bayesian analyses, we ran frequentist analysis using more conventional mixed models. These produced a corresponding pattern of results and can be seen in the SI.

## Results

As predicted, in the individual payoff condition, participants conformed to each other’s responses, lowering their diversity and drifting away from the correct answer. In the collective payoff condition, when social information was present, participants’ responses remained equally diverse and with their accuracy unaffected than when social information was absent (Fig 2). Full tables of parameter estimates and contrasts between conditions are given in the SI (Group diversity: S1–S6 Tables; Group error: S7–S9 Tables).

For group diversity overall, we found no strong evidence that payoff conditions differed from each other (MPE = 76.25%). In other words, there was not a high chance that the difference between collective and individual payoff conditions was greater than zero. There was an overall main effect of social information, however (MPE = 95.85%), with lower diversity in responses when social information was present. As can been seen in Fig 2, this difference in social information is driven by differences within the individual payoff conditions.

We quantified the strength of evidence for an effect of social information within each of the payoff conditions separately. For group diversity in the individual payoff condition, there was a very high probability (MPE = 99.88%) that there is less diversity in responses when social information was present. There was little evidence (MPE = 73.35%) of an effect of social information in the collective payoff condition.

For group error, there was not a strong probability (MPE = 65.35%) that payoff conditions differed from each other overall. However, there was a main effect of social information condition (MPE = 98.32%) with greater errors when social information was present. Once more, this difference appeared to be driven by an effect of social information within the individual pay off condition. There was strong evidence (MPE = 99.57%) that group error was higher when social information was present, but little evidence (MPE = 69.13%) that it played a role in the collective condition.

## Discussion

We investigated how the structure of incentives and the availability of social information affect social conformity and determine collective intelligence. We found that when people are rewarded as individuals, they are, counter-intuitively, more likely to act as a herd by conforming more when social information is available. This reduces the diversity of opinions in the group and decreases the overall accuracy of the collective estimate. When people are rewarded as a collective, individuals are unaffected by social information, and diversity and group wisdom is maintained. Our study demonstrates that incentive structure has important consequences for group dynamics, modifying the way individuals respond to social information.

Groups of individuals can be strikingly accurate at estimating answers to problems where the individual members may have little or no knowledge [2]. However, consistent with previous work [35], we observed that knowledge of others’ responses undermines the wisdom of the crowd by increasing conformity and narrowing the diversity of opinions.

Our result seems counter-intuitive, in the context of how we typically think of motivation and collective behaviour outside of the laboratory. One of the tenets of political economics is the assumption that “every-man-for-himself” payoff incentives are potent motivators of diversity which promote individuals to defy their societal norms [27]. Our results show the opposite. Consistent with the increase in conformity we observed here, several other studies have suggested that when making decisions under uncertainty, following others may be advisable for various reasons: others might know something we do not know; we may be held less responsible of our conformist choices; and making a mistake individually may be very costly [20,36,37].

Some studies on problem solving tasks have shown that providing social information actually improves group-level performance [38]; copying others allows group members to converge to a “good enough” solution quickly rather than searching for innovative solutions that might be risky or redundant [39]. The tendency to copy others’ opinions depends on individual differences (some are more pliable than others), and how different one’s opinion is relative to the group—individuals tend to adjust their opinion if it is very different from the group than if it is similar [38]. Here we demonstrate that incentive structure also affects how individuals make decisions in response to social information.

In individually centred situations, a person would be less concerned about how others’ opinions affect the group result, because they are only rewarded for their own response. Copying others is beneficial because of the increased information available. Therefore under individual payoff conditions, social influence narrows diversity of opinions, through copying behaviour, and reduces variation. In contrast, rewarding individuals for the accuracy of their group’s performance counter-intuitively, promotes more diversity of opinions—hence a reduction in herding behaviour. Here, individuals are more likely to consider the group outcome and how their own, and others’, opinions contribute to it (since their reward depends on it). Thus to maximise group performance, diversifying opinions is better than copying.

Our findings suggest that the trade-off between individuality, information and motivation is fundamental to understanding collective behaviour and can have profound implications for a broad range of social phenomena. For example, a collective incentive structure that may actually promote liberal values and encourage individual differences will likely have a non-trivial effect in a democratic community.

In this experiment, for simplicity, we provided social information as an aggregation of information (the group mean). Therefore participants observed a post hoc snapshot view of the group opinion. We did not display the participants’ individual answers to each other, which would require group members to accumulate the social information themselves, or individuals’ level of confidence in their answers, which would enable others to judge the importance of their decisions [40]. These factors, in addition to any kind of social psychological influence, such as leader effects and persuasion, all add increased complexity to the system. Therefore their effect on the individual, and consequently group, response should be explored further in future studies.

The idea of an independent individual often forms the basis of concepts in economics and cognitive sciences, and the notion of individualism and rational choice has profoundly shaped Western culture. However people interact in social networks, which can lead to the co-evolution of behaviours and social relationships—people make connections and influence one another [41], affecting the degree to which they truly act as independent individuals. Our results demonstrate that this can also be influenced by incentive structure, where individuality is reduced when individuals are acting in their own interests.

## Supporting information

S1 Fig Questions and answers. A full list of questions asked during experimental trials and the corresponding answers shown on the map of London.
(PDF)

S1 Video Video animation showing an example trial. A video showing an example trial for the individual payoff and social information was present conditions. The video shows the steps within a trial as outlined in Fig 2. Blue dots show participant responses. The dots are displayed in the video to show participants’ decision-making in real-time but were not visible to participants during the experiments. Participants were only able to see their own dot on their device or the star (the mean group response) on the shared display in the social information present condition.
(M4V)

S1 File Results—Bayesian mixed models and frequentist analyses. Detailed statistics results of the Bayesian Mixed Models and Frequentist analyses.
(DOCX)

S2 File Trial data for error and diversity measures. The data that support the findings of this study.
(XLSX)

S1 Table Bayesian mixed model group diversity estimates for each model parameter. (DOCX)

S2 Table Bayesian mixed model estimates of the group diversity at different levels of the experimental conditions. (DOCX)

S3 Table Quantifying the evidence for the contrasts between experimental conditions using Bayesian mixed models for group diversity. (DOCX)

S4 Table Bayesian mixed model group diversity estimates for each model parameter using the distribution of final responses alone. (DOCX)

S5 Table Bayesian mixed model estimates of the group diversity at different levels of the experimental conditions using the distribution of final responses alone. (DOCX)

S6 Table Quantifying the evidence for the contrasts between experimental conditions using Bayesian mixed models using the distribution of final responses alone. (DOCX)

S7 Table Bayesian mixed model group error estimates for each model parameter. (DOCX)

S8 Table Bayesian mixed model estimates of the group error at different levels of the experimental conditions. (DOCX)

S9 Table Quantifying the evidence for the contrasts between experimental conditions using Bayesian mixed models for group error. (DOCX)

## References

[1] Krause J, Ruxton G. D., Krause S (2009) Swarm intelligence in animals and humans. Trends in Ecology and Evolution 25: 28–34. 10.1016/j.tree.2009.06.016 19735961

[2] Surowiecki J (2004) The Wisdom of the Crowds: Why the Many are Smarter than the Few: Knopf Doubleday Publishing Group 336 p.

[3] Arrow KJ, Forsythe R, Gorham M, Hahn R, Hanson R, Ledyard J, et al (2008) The Promise of Prediction Markets. Science 320: 877–878. 10.1126/science.1157679 18487176

[4] Wolfers J, Zitzewitz E (2004) Prediction Markets. Journal of Economic Perspectives 18: 107–126.

[5] Galton F (1907) Vox populi. Nature 75: 450–451.

[6] Page SE (2008) The Difference: How the Power of Diversity Creates Better Groups, Firms, Schools, and Societies. Princeton: Princeton University Press.

[7] King AJ, Cheng L, Starke SD, Myatt JP (2011) Is the true ‘wisdom of the crowd’ to copy successful individuals? Biology Letters 8: 197–200. 10.1098/rsbl.2011.0795 21920956 PMC3297389

[8] Lorge I, Fox D, Davitz J, Brenner M (1958) A survey of studies contrasting the quality of group performance and individual performance. Psychological Bulletin 55: 337–372. 10.1037/h0042344 13602018

[9] Yaniv I, Milyavsky M (2007) Using advice from multiple sources to revise and improve judgments. Organizational Behavior and Human Decision Processes 103: 104–120.

[10] Kaplan C, A. (2001) Collective Intelligence: A new approach to stock price forecasting. Proceedings of the 2001 IEEE Systems, Man, and Cybernetics Conference: IEEE.

[11] Malone TW, Klein M (2007) Harnessing Collective Intelligence to Address Global Climate Change. Innovations 2: 15–26.

[12] Lih A (2009) The Wikipedia Evolution: How a Bunch of Nobodies Created the World’s Greatest Encyclopedia. New York: NY: Hyperion.

[13] Janis IL (1972) Victims of Groupthink: a Psychological Study of Foreign-Policy Decisions and Fiascoes.: Boston: Houghton Mifflin.

[14] Park J, Konana PC, Gu B, Kumar A, Raghunathan R (2013) Information valuation and confirmation bias in virtual communities: evidence from stock message boards. Information Systems Research 24: 1050–1067.

[15] Barsade SG (2002) The Ripple Effect: Emotional Contagion and its Influence on Group Behavior. Administrative Science Quarterly 47: 644–675.

[16] Mackay C (1852) Extraordinary Popular Delusions and the Madness of Crowds. London, UK.

[17] Asch SE (1955) Opinions and Social Pressure. Scientific American 193: 31–35.

[18] Christakis N, Fowler J (2009) Connected: The Amazing Power of Social Networks and How They Shape Our Lives: Little, Brown and Company.

[19] Raafat R. M., Charter N, Frith C (2009) Herding in humans. Trends in Cognitive Sciences 13: 420–428. 10.1016/j.tics.2009.08.002 19748818

[20] Farrell S (2011) Social influence benefits the wisdom of individuals in the crowd. Proceedings of the National Academy of Sciences 108: E625. 10.1073/pnas.1109947108 PMC3169111 21876181

[21] Lichtendahl KC, Grushka-Cockayne Y, Pfeifer PE (2013) The wisdom of competitive crowds. Darden Business School Working Paper No 1926330.

[22] Pfeifer PE (2015) The promise of pick-the-winners contests for producing crowd probability forecasts. Theory and Decision: 1–24.

[23] Pfeifer PE, Grushka-Cockayne Y, Lichtendahl KC (2014) The promise of prediction contests. The American Statistician 68: 264–270.

[24] Hong L, Page SE, Riolo M (2012) Incentives, information, and emergent collective accuracy. Managerial and Decision Economics 33: 323–334.

[25] Mann RP, Helbing D (2017) Optimal incentives for collective intelligence. Proceedings of the National Academy of Sciences published ahead of print May 1, 2017. 10.1073/pnas.1618722114 PMC5441831 28461491

[26] Sumpter DJT (2006) The principles of collective animal behaviour. Philosophical Transactions of the Royal Society of London: Series B 361: 5–22. 16553306 10.1098/rstb.2005.1733 PMC1626537

[27] Smith A (1759) The Theory of Moral Sentiments: London: A. Millar.

[28] Gale D, Kariv S (2003) Bayesian Learning in Social Networks. Games and Economic Behavior 45: 329–346.

[29] von Zimmermann J, Richardson D (in prep) The Hive—https://thehive.sc/welcome.

[30] Kruschke JK (2010) Bayesian data analysis. Wiley Interdisciplinary Reviews: Cognitive Science 1: 658–676. 10.1002/wcs.72 26271651

[31] Wagenmakers EJ, Wetzels R, Borsboom D, van der Maas HLJ (2011) Why psychologists must change the way they analyze their data: The case of psi: The case of psi: Comment on Bem. Journal of Personality and Social Psychology 100: 426–432. 10.1037/a0022790 21280965

[32] R-Core-Team (2017) R: A language and environment for statistical computing. R Foundation for Statistical Computing. Vienna, Austria.

[33] Stan-Development-Team (2016) rstanarm: Bayesian applied regression modeling via Stan. R package version 2.13.1. http://mc-stan.org/.

[34] Makowski (2018) The psycho Package: an Efficient and Publishing-Oriented Workflow for Psychological Science. Journal of Open Source Software 3: 470.

[35] Lorenz J, Rauhut H, Schweitzer F, Helbing D (2011) How social influence can undermine the wisdom of crowd effect. Proceedings of the National Academy of Sciences 108: 9020–9025. 10.1073/pnas.1008636108 PMC3107299 21576485

[36] Bentley RA, Brock WA, Caiado CCS, O'Brien MJ (2016) Evaluating reproductive decisions as discrete choices under social influence. Philosophical Transactions of The Royal Society B: Biological Sciences 371: 20150154. 10.1098/rstb.2015.0154 PMC4822434 27022081

[37] Danchin E, Giraldeau L-A, Valone T. J., Wagner R. H. (2004) Public Information: From Nosy Neighbors to Cultural Evolution. Science 305: 487–491. 10.1126/science.1098254 15273386

[38] Granovskiy B, Gold JM, Sumpter DJT, Goldstone RL (2015) Integration of Social Information by Human Groups. Topics in Cognitive Science 7: 469–493. 10.1111/tops.12150 26189568 PMC4545507

[39] Wisdom TN, Song X, Goldstone RL (2013) Social Learning Strategies in Networked Groups. Cognitive Science 37: 1383–1425. 10.1111/cogs.12052 23845020

[40] Bahrami B, Olsen K, Latham P, Roepstorff A, Rees G, Frith C (2010) Optimally interacting minds. Science 329: 1081–1085. 10.1126/science.1185718 20798320 PMC3371582

[41] Dong W, Lepri B, Pentland A. Modeling the Co-evolution of Behaviors and Social Relationships Using Mobile Phone Data; 2011; Beijing, China. pp. 134–143.

