# Supplementary material for: Self-serving incentives impair collective decisions by increasing conformity
Source: PLoS One. 2019 Nov 14;14(11):e0224725. doi: 10.1371/journal.pone.0224725 (PMC6855459; doi:10.1371/journal.pone.0224725)

Where did Oscar Wilde live?

Where was Edward VI born?

Where was H.G. Wells born?

Where were the first bombs dropped in WWI?

Where did Arthur Conan Doyle live in 1891?

Where has the most number of violent crimes?

Where is the UK McDonald’s headquarters?

Where was David Beckham born?

Where was Alan Turing born?

Where the oldest living tree?

Where was Jack The Ripper’s first victim found?

Where were The Rolling Stones discovered?

Where was Elizabeth I born?

Where were the first escalators installed?

Where did William Morris live in 1860?

Where are the most Koreans?

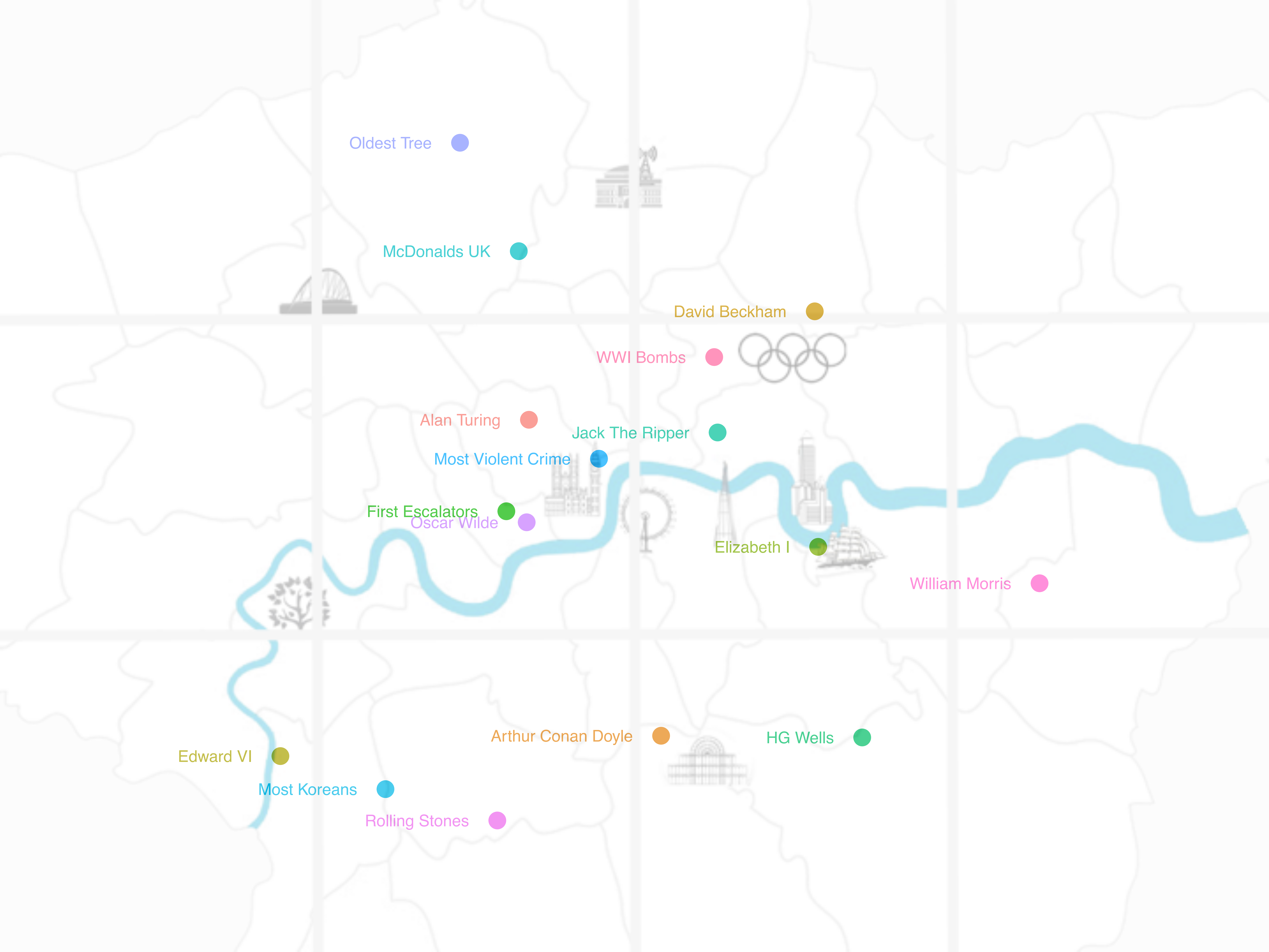

Supplement: S1 Fig — A full list of questions asked during experimental trials and the corresponding answers shown on the map of London. (PDF) [file pone.0224725.s001.pdf]
