# Supplementary material for: Self-serving incentives impair collective decisions by increasing conformity
Source: PLoS One. 2019 Nov 14;14(11):e0224725. doi: 10.1371/journal.pone.0224725 (PMC6855459; doi:10.1371/journal.pone.0224725)
Supplement: S1 File — Detailed statistics results of the Bayesian Mixed Models and Frequentist analyses. (DOCX) [file pone.0224725.s003.docx]

**Supplementary Information**

**Bayesian Mixed Models Results**

Here we report detailed statistics for each model parameter of interest.

**Group Diversity**

We fitted a Markov Chain Monte Carlo model to distances between each individual dot and the group average, with random effects for question and group. The model was specified as:

diversity ~ socialinfo * payoff + (1 | question ) + (1 | group ) + (1 | participant )

Weakly informative priors from the Gaussian family were used that were scaled by the rstanarm package:

Intercept (after predictors centered)

~ normal(location = 0, scale = 10)

**adjusted scale = 1.33

Coefficients

~ normal(location = [0,0,0], scale = [2.5,2.5,2.5])

**adjusted scale = [0.33,0.33,0.33]

Auxiliary (sigma)

~ exponential(rate = 1)

**adjusted scale = 0.13 (adjusted rate = 1/adjusted scale)

Covariance

~ decov(reg. = 1, conc. = 1, shape = 1, scale = 1)

Estimates of model parameters can be found in S1 Table. We used the psycho package and emmeans to calculate estimates of the group diversity at different levels of the experimental conditions (S2 Table), and to quantify the evidence for these contrasts between conditions (S3 Table).

**Group Diversity – Final response**

Using the same model as above, we also analysed diversity of responses using the distribution of final responses alone. These results gave the same pattern of results.

Estimates of model parameters can be found in S4 Table, estimates of the group diversity at different levels of the experimental conditions can be found in S5 Table, and evidence for contrasts between conditions can be found in S6 Table.

**Group Error**

A similar process was followed for group error. We fitted a Markov Chain Monte Carlo model to distances between each individual dot and the group average, with random effects for question and group. The model was specified as:

grouperror ~ socialinfo * payoff + (1 | question ) + (1 | group )

Weakly informative priors from the Gaussian family were used that were scaled by the rstanarm package:

Intercept (after predictors centered)

~ normal(location = 0, scale = 10)

**adjusted scale = 1.41

Coefficients

~ normal(location = [0,0,0], scale = [2.5,2.5,2.5])

**adjusted scale = [0.35,0.35,0.35]

Auxiliary (sigma)

~ exponential(rate = 1)

**adjusted scale = 0.14 (adjusted rate = 1/adjusted scale)

Covariance

~ decov(reg. = 1, conc. = 1, shape = 1, scale = 1)

Estimates of model parameters can be found in S7 Table, estimates of the group diversity at different levels of the experimental conditions can be found in S8 Table, and evidence for contrasts between conditions can be found in S9 Table.

**Frequentist analyses**

We also analysed our data with more conventional mixed models and ANOVAs. These produced corresponding patterns of results as our Bayesian analyses.

**Group Diversity**

To account for the fact that individuals were nested within groups we employed a generalised linear mixed model, with fixed effects for social information and reward conditions, and random effects for the question, the group and individuals, each with random intercepts. The Kenward-Roger approximation for degrees-of-freedom method was used to derive p-values. There was a significant interaction between social information and payoff condition (*F*(1,2087.47)=6.60, *p*=0.01) and non-significant main effects of payoff conditions (*F*(1,2093.79)=0.53, *p*=0.47) and social information (*F*(1,2094.78)=3.01, *p*=0.08). This pattern of results was mirrored by an 2x2 ANOVA analysis that found a significant interaction between social information and payoff condition (*F*(1,139)=6.19, *p*=0.01), and non-significant effect of payoff (*F*(1,39)=1.19, *p*=0.28) and social information condition (*F*(1,139)=2.36, *p*=0.13).

We carried out planned comparisons on the effect of social information in each payoff condition. T-tests showed an effect of social information in the individual payoff condition (*t*(139)=-2.90, *p*=0.004) but not the collective (*t*(139)=0.54, *p*=0.59).

**Group error**

Error was calculated for each group as the distance between the group mean position and the correct answer on each trial. We employed a generalised linear mixed model with fixed effects for social information and reward conditions, and random effects for the group and the question item with random intercepts. There was a significant effect of social information condition (*F*(1,327.25)=4.52, *p*=0.03), and non-significant effect of payoff conditions (*F*(1,327.25)=0.16, *p*=0.69) and interaction (*F*(1,327.03)=2.02, *p*=0.16). This pattern of results was mirrored by an 2x2 ANOVA analysis that found a significant effect of social information condition (*F*(1,22)=5.51, *p*=0.03), and non-significant effect of payoff conditions (*F*(1,22)=0.38, *p*=0.54) and interaction (*F*(1,22)=1.82, *p*=0.16).

We carried out planned comparisons on the effect of social information in each payoff condition as above. T-tests, showing an effect of social information in the individual payoff condition (*t*(22)=-2.84, *p*=0.01) but not the collective (*t*(22)=0.58, *p*=0.57).
