# Supplementary material for: Self-serving incentives impair collective decisions by increasing conformity
Source: PLoS One. 2019 Nov 14;14(11):e0224725. doi: 10.1371/journal.pone.0224725 (PMC6855459; doi:10.1371/journal.pone.0224725)
Supplement: S1 Table — (DOCX) [file pone.0224725.s005.docx]

**S1 Table. Bayesian mixed model group diversity estimates for each model parameter**

| **Variable** | **MPE** | **Median** | **MAD** | **95 CI**  **lower** | **95 CI**  **upper** |
| --- | --- | --- | --- | --- | --- |
| (Intercept) | 0 | 0.212 | 0.013 | 0.186 | 0.238 |
| socialInfo=present | 71.4 | 0.004 | 0.007 | -0.009 | 0.02 |
| Payoff=Individual | 90.38 | 0.009 | 0.007 | -0.005 | 0.023 |
| socialInfo=present X Payoff=Individual | 99.58 | -0.026 | 0.01 | -0.047 | -0.006 |
