# Supplementary material for: Self-serving incentives impair collective decisions by increasing conformity
Source: PLoS One. 2019 Nov 14;14(11):e0224725. doi: 10.1371/journal.pone.0224725 (PMC6855459; doi:10.1371/journal.pone.0224725)
Supplement: S2 Table — (DOCX) [file pone.0224725.s006.docx]

**S2 Table. Bayesian mixed model estimates of the group diversity at different levels of the experimental conditions**

| **Social information** | **Payoff** | **Median** | **MAD** | **95 CI**  **lower** | **95 CI**  **upper** |
| --- | --- | --- | --- | --- | --- |
| absent | collective | 0.212 | 0.013 | 0.191 | 0.234 |
| present | collective | 0.216 | 0.012 | 0.195 | 0.237 |
| absent | individual | 0.221 | 0.013 | 0.2 | 0.243 |
| present | individual | 0.199 | 0.012 | 0.178 | 0.22 |
