# Supplementary material for: Self-serving incentives impair collective decisions by increasing conformity
Source: PLoS One. 2019 Nov 14;14(11):e0224725. doi: 10.1371/journal.pone.0224725 (PMC6855459; doi:10.1371/journal.pone.0224725)
Supplement: S3 Table — (DOCX) [file pone.0224725.s007.docx]

**S3 Table. Quantifying the evidence for the contrasts between experimental conditions using Bayesian mixed models for group diversity**

| **Contrast** | **MPE** | **Median** | **MAD** | **95 CI**  **lower** | **95 CI**  **upper** |
| --- | --- | --- | --- | --- | --- |
| Main effect:  Social information absent VS present | 95.85 | 0.009 | 0.005 | 0.001 | 0.017 |
| Main effect:  Payoff collective VS individual | 76.25 | 0.004 | 0.005 | -.005 | 0.012 |
| Payoff = collective  Social information absent VS present | 73.35 | -0.004 | 0.007 | -0.015 | 0.007 |
| Payoff = individual  Social information absent VS present | 99.82 | 0.022 | 0.007 | 0.010 | 0.034 |
