# Supplementary material for: Self-serving incentives impair collective decisions by increasing conformity
Source: PLoS One. 2019 Nov 14;14(11):e0224725. doi: 10.1371/journal.pone.0224725 (PMC6855459; doi:10.1371/journal.pone.0224725)
Supplement: S4 Table — (DOCX) [file pone.0224725.s008.docx]

**S4 Table. Bayesian Mixed Model group diversity estimates for each model parameter using the distribution of final responses alone**

| **Variable** | **MPE** | **Median** | **MAD** | **95 CI**  **lower** | **95 CI**  **upper** |
| --- | --- | --- | --- | --- | --- |
| (Intercept) |  | 0.203 | 0.013 | 0.177 | 0.226 |
| socialInfo=present | 62.32 | -.0.002 | 0.007 | -0.016 | 0.012 |
| Payoff=Individual | 78.57 | 0.006 | 0.007 | -0.008 | 0.019 |
| socialInfo=present X Payoff=Individual | 98.80 | -0.022 | 0.009 | -0.042 | -0.003 |
