# Supplementary material for: Self-serving incentives impair collective decisions by increasing conformity
Source: PLoS One. 2019 Nov 14;14(11):e0224725. doi: 10.1371/journal.pone.0224725 (PMC6855459; doi:10.1371/journal.pone.0224725)
Supplement: S6 Table — (DOCX) [file pone.0224725.s010.docx]

**S6 Table. Quantifying the evidence for the contrasts between experimental conditions using Bayesian mixed models using the distribution of final responses alone**

| **Contrast** | **MPE** | **Median** | **MAD** | **95 CI**  **lower** | **95 CI**  **upper** |
| --- | --- | --- | --- | --- | --- |
| Main effect:  Social information absent VS present | 99.52 | 0.013 | 0.005 | 0.005 | 0.021 |
| Main effect:  Payoff collective VS individual | 87.02 | 0.005 | 0.005 | -.003 | 0.013 |
| Payoff = collective  Social information absent VS present | 62.32 | 0.002 | 0.007 | -0.009 | 0.013 |
| Payoff = individual  Social information absent VS present | 99.95 | 0.024 | 0.007 | 0.012 | 0.035 |
