# Supplementary material for: Self-serving incentives impair collective decisions by increasing conformity
Source: PLoS One. 2019 Nov 14;14(11):e0224725. doi: 10.1371/journal.pone.0224725 (PMC6855459; doi:10.1371/journal.pone.0224725)
Supplement: S7 Table — (DOCX) [file pone.0224725.s011.docx]

**S7 Table. Bayesian mixed model group error estimates for each model parameter**

| **Variable** | **MPE** | **Median** | **MAD** | **95 CI**  **lower** | **95 CI**  **upper** |
| --- | --- | --- | --- | --- | --- |
| (Intercept) | 0 | 0.284 | 0.03 | 0.224 | 0.349 |
| socialInfo | 69.98 | 0.007 | 0.013 | -0.017 | 0.03 |
| payoff | 75.03 | -0.009 | 0.013 | -0.032 | 0.016 |
| socialInfo X payoff | 91.83 | 0.024 | 0.017 | -0.011 | 0.057 |
