# Supplementary material for: Self-serving incentives impair collective decisions by increasing conformity
Source: PLoS One. 2019 Nov 14;14(11):e0224725. doi: 10.1371/journal.pone.0224725 (PMC6855459; doi:10.1371/journal.pone.0224725)
Supplement: S8 Table — (DOCX) [file pone.0224725.s012.docx]

**S8 Table. Bayesian mixed model estimates of the group error at different levels of the experimental conditions**

| **Social information** | **Payoff** | **Median** | **MAD** | **95 CI**  **lower** | **95 CI**  **upper** |
| --- | --- | --- | --- | --- | --- |
| absent | collective | 0.285 | 0.032 | 0.23 | 0.342 |
| present | collective | 0.292 | 0.032 | 0.235 | 0.345 |
| absent | individual | 0.277 | 0.032 | 0.222 | 0.332 |
| present | individual | 0.307 | 0.033 | 0.252 | 0.364 |
