# Supplementary material for: Self-serving incentives impair collective decisions by increasing conformity
Source: PLoS One. 2019 Nov 14;14(11):e0224725. doi: 10.1371/journal.pone.0224725 (PMC6855459; doi:10.1371/journal.pone.0224725)
Supplement: S9 Table — (DOCX) [file pone.0224725.s013.docx]

**S9 Table. Quantifying the evidence for the contrasts between experimental conditions using Bayesian mixed models for group error**

| **Contrast** | **MPE** | **Median** | **MAD** | **95 CI**  **lower** | **95 CI**  **upper** |
| --- | --- | --- | --- | --- | --- |
| Main effect:  Social information absent VS present | 98.32 | -0.019 | 0.009 | -0.034 | -0.005 |
| Main effect:  Payoff collective VS individual | 65.53 | -0.004 | 0.009 | -0.018 | 0.010 |
| Payoff = collective  Social information absent VS present | 69.13 | -0.006 | 0.012 | -0.028 | 0.013 |
| Payoff = individual  Social information absent VS present | 99.57 | -0.031 | 0.012 | -0.051 | -0.010 |
